# Knowledge and attitudes toward e-cigarette smoking among health and non-health science students: A cross-sectional study

**DOI:** 10.18332/tpc/215587

**Published:** 2026-05-19

**Authors:** Prasittichai Poonphol, Pharanyou Lueamsingkhon, Tiwaphon Thongsutt, Tuanthon Boonlue

**Affiliations:** 1 Department of Pharmacy Practice, Faculty of Pharmaceutical Sciences, Ubon Ratchathani University, Ubon Ratchathani, Thailand

**Keywords:** e-cigarettes, knowledge, attitudes, university students, health sciences, Thailand

## Abstract

**INTRODUCTION:**

E-cigarettes are increasingly popular among university students, raising significant public health concerns. This study aimed to assess the prevalence of e-cigarette use, as well as knowledge, and attitudes toward e-cigarettes among health and non-health science undergraduate students in Thailand.

**METHODS:**

A cross-sectional survey among health and non-health science students in Thailand was conducted between March and September 2023 using a self-administered online questionnaire. Data were analyzed using descriptive statistics, chi-squared tests, and multivariate logistic regression.

**RESULTS:**

Among 440 participants, 37.05% were from health science programs and 62.95% from non-health science. E-cigarette use was reported by 15.7% of health science and 27.7% of non-health science students (p=0.001). Significant differences were also found in income (p<0.001), family smoking history (p=0.006), and alcohol consumption (p<0.001). Health science students had greater awareness of health risks, including lung inflammation (91.4% vs 85.9%, p=0.04, AOR=0.69; 95% CI: 0.47–0.98) and lung cancer (90.8% vs. 81.6%, p=0.006, AOR=1.62; 95% CI: 1.14–2.28). In contrast, non-health science students were more likely to believe e-cigarettes aid smoking cessation (39.4% vs 21.5%, p=0.02, AOR=1.36; 95% CI: 1.05–1.77). Despite these differences, misconceptions about nicotine addiction and safety remained common across both groups.

**CONCLUSIONS:**

The findings highlight the need for targeted educational programs and public health interventions to address misconceptions and discourage e-cigarette use among university students. Improved health education is essential to equip students with accurate information for making informed decisions about e-cigarettes.

## INTRODUCTION

The global public health debate and research interest have been significantly impacted by the rapid proliferation of electronic cigarettes (e-cigarettes). E-cigarettes have become increasingly popular among various demographics, with young adults and university students among the most enthusiastic advocates^1^. In recent years, surveys have indicated that over 3.6% of adults and 10–20% of adolescents have used e-cigarettes, indicating a substantial increase in the global prevalence of e-cigarette usage^2^. Despite the ongoing debates regarding the long-term health implications of e-cigarettes, the belief that they are a less harmful alternative to conventional smoking is partially responsible for the increase in their utilization^3,4^.

E-cigarettes include nicotine, a substance that is highly addictive and may interfere with adolescents’ brain development, resulting in ongoing cognitive and behavioral problems^5^. E-cigarette vapor may contain substances such as formaldehyde and acetaldehyde, which can adversely affect respiratory and cardiovascular health^6-9^. These hazards underscore the necessity for additional study and education.

Thailand has implemented one of the most comprehensive regulatory frameworks prohibiting electronic nicotine delivery systems (ENDS), including both liquid e-cigarettes and heated tobacco products. In 2014, the Ministry of Commerce issued a ban on the importation of ENDS under the Export and Import of Goods Act, prescribing penalties of up to 10 years of imprisonment and fines equivalent to five times the value of the imported goods. Subsequently, in 2015, the Office of the Consumer Protection Board prohibited the domestic sale of these products, with violators subject to a maximum of five years’ imprisonment and fines up to 500000 THB. The Tobacco Products Control Act, revised in 2017, formally classified ENDS as tobacco products, thereby extending restrictions on their use in public spaces and prohibiting all forms of advertising and promotion^10^. Despite these legislative efforts, enforcement challenges remain, particularly regarding the proliferation of ENDS through illicit and online markets. These regulatory gaps may contribute to persistent misconceptions and limited access to evidence-based cessation support among young adults.

University students represent a crucial group for studying e-cigarette knowledge and attitudes, as they are in a transitional phase shaped by social and behavioral influences. Studies have demonstrated differing levels of awareness and misunderstandings worldwide^11-13^. In the United States, numerous college students recognized e-cigarettes but underestimated their health hazards, frequently perceiving them as less detrimental than conventional cigarettes^14^. Previous studies in Thailand have highlighted gaps in public understanding of e-cigarettes and limited access to cessation resources, which may reflect challenges in health communication and enforcement of existing regulations. These findings underscore the importance of integrating effective educational strategies and accessible cessation services within the broader regulatory framework^15-17^.

In contrast to Thailand, where e-cigarettes are strictly prohibited in terms of importation, sale, and use, the United Kingdom adopts a more permissive approach. The UK has integrated e-cigarettes into its harm reduction strategy, allowing their regulated sale and marketing as an alternative for smoking cessation^18^. Public Health England has endorsed e-cigarettes as a less harmful option compared to conventional cigarettes, supported by evidence suggesting they are 95% less harmful than smoking when used under regulated conditions^18^. Moreover, strict regulations in the UK ensure product safety and quality while discouraging use among non-smokers and youth . This divergent regulatory approach reflects differences in public health priorities, with Thailand emphasizing outright bans to deter youth access, and the UK focusing on harm reduction for smokers. These contrasting frameworks provides an interesting comparison into how regulation shapes public perceptions and use of e-cigarettes.

This study aims to evaluate the knowledge and attitudes of e-cigarettes among Thai undergraduate students via a cross-sectional survey. The findings may enhance comprehension of e-cigarette beliefs by identifying information gaps and prevailing attitudes, so informing targeted public health policies and educational initiatives for university students.

## METHODS

This cross-sectional survey study gathered data through self-administered online questionnaires regarding knowledge and attitudes of e-cigarettes among Thai undergraduate students, conducted from 1 March to 30 September 2023. We included all of university students in Ubon Ratchathani University in academic year 2023. Participants who did not complete the online questionnaire or declined to provide consent were excluded from the study.

### Sample size

The minimum required sample size was determined using a total population size of 16723, a population proportion of 0.20 (according to the study by Kochsiripong and Pitirattanaworranat^19^), and a margin of error of 0.05. Based on these parameters, the minimum sample size required for this study was 243 participants. To achieve this, a database of Ubon Ratchathani University students was obtained from the university’s registration office.

This study employed a stratified sampling technique based on academic program groups at Ubon Ratchathani University. The university comprises 11 faculties, categorized into three main groups: 1) Arts, Humanities, and Social Sciences, which includes faculties such as Business School, Law, Liberal Arts, and Political Science; 2) Science and Technology, encompassing faculties such as Agriculture, Applied Arts and Architecture, Engineering, and Science; and 3) Health Sciences, including faculties such as Medicine and Public Health, Nursing, and Pharmaceutical Sciences. A stratified sampling technique was employed to ensure adequate representation of students from both health and non-health science faculties. The university’s student population was divided into three main groups based on academic disciplines: health sciences, sciences and technology, and arts, humanities, and social sciences. Proportional stratification was used, with the sample size for each group determined relative to their representation within the total student population. This approach ensured that the health science group and the non-health science group (comprising the other two categories) were adequately represented in the study. Stratification aimed to capture the diversity of knowledge, attitudes, and behaviors regarding e-cigarette use across academic disciplines, allowing for meaningful comparisons between the groups. The distribution of the questionnaire was facilitated through the university’s student affairs personnel, who disseminated the survey via email or an online platform. The Human Research Ethics Committee of Ubon Ratchathani University authorized the study protocol (UBU-REC-52/2566).

### Measures and data collection

The questionnaire was only available in Thai. The questionnaire was formulated and constructed based on the literature from E-cigarette Knowledge, Beliefs and Attitude Questionnaire^15^. The questionnaire was developed based on the study findings and further refined with the assistance of at least three experts who assessed the content validity. The questionnaire was assessed by four specialists, comprising two pharmacy lecturers who teach research methodology, one hospital pharmacist and one community pharmacist who have relevant experience in tobacco control and cessation, all of whom have experience in evaluating survey items. Each expert rated the relevance of each item on the questionnaire using a scale of -1, 0, and 1. These ratings were used to calculate the Index of Item Objective Congruence (IOC) for each item. An IOC score of 0.5 or higher was considered acceptable. In this study, the IOC score was 0.84, indicating that the items had strong content validity and were aligned with the research objectives. Additionally, the Cronbach’s alpha for the questionnaire was calculated to be 0.793, demonstrating a good level of internal consistency for the items.

The final version of the questionnaire comprised three sections. The first section gathered sociodemographic data, including age, gender, faculty, and other relevant characteristics. The second section consisted of closed-ended questions designed to assess participants’ knowledge about e-cigarettes. Participants were asked to respond to questions regarding the legality, safety, and health implications of e-cigarette use. The third section evaluated participants’ attitudes towards e-cigarettes using a two-point Likert scale (agree/disagree).

All participants were provided with written information outlining the study objectives, procedures, voluntary participation, and data confidentiality. Informed consent was obtained prior to questionnaire completion. Data were collected electronically via Google Forms and exported to Microsoft Excel for analysis. Responses were anonymized, and all data were securely stored with restricted access to the research team.

### Data analysis

Descriptive statistics were used to present all study variables. Continuous variables were presented as means and standard deviations (or medians and interquartile ranges, where appropriate), while categorical variables were reported as frequencies and percentages. Univariate logistic regression analysis was conducted to assess differences between the health science and non-health science groups. Variables with p<0.1 in the univariate analysis were included in the multivariate logistic regression model. Statistical significance was set at p<0.05. All data analyses were performed using Stata Version 14.

## RESULTS

Table 1 summarizes the baseline characteristics of the 440 participants, with 37.05% from health science faculties and 62.95% from non-health science faculties. Significant differences were observed between the groups in terms of age, income, family smoking history, alcohol use, and e-cigarette use.

**Table 1:** Sociodemographic characteristics and e-cigarette use among university students, a cross-sectional study at Ubon Ratchathani University, Thailand, 2023 (N=440)

*Characteristics*	*Health science* *(N=163)* *n (%)*	*Non-health science* *(N=277)* *n (%)*	p*
**Gender**			
Male	41 (25.15)	82 (29.60)	0.32
Female	122 (74.85)	195 (70.40)	
**Age** (years)			
18–19	24 (14.72)	61 (22.02)	<0.001
20–21	38 (23.31)	92 (33.21)	
22–23	57 (34.97)	105 (37.91)	
>23	44 (26.99)	19 (6.86)	
**Income per month** (THB)			
<5000	29 (17.79)	89 (32.13)	<0.001
5001–10000	83 (50.92)	148 (53.43)	
10001–15000	36 (22.09)	31 (11.19)	
>15000	15 (9.20)	9 (3.25)	
**History of smoking in family**			
No	110 (67.48)	150 (54.15)	0.006
Yes	53 (32.52)	127 (45.85)	
**Alcohol use**			
No	45 (27.61)	30 (10.83)	<0.001
Yes	118 (72.39)	240 (89.17)	
**E-cigarette use**			
No	138 (84.66)	194 (70.04)	0.001
Yes	25 (15.34)	83 (29.96)	

* Chi-squared tests. THB: 1000 Thai Baht about US$32.

Health science students were older, with a mean age of 22.42 ± 3.39 years compared to 21.10 ± 1.86 years for non-health science students (p<0.001). A greater proportion of health science students were aged ≤22 years (61.96%), whereas the majority of non-health science students were younger than 22 years (55.23%) (p<0.001).

Monthly income distribution also differed significantly (p<0.001), with more non-health science students earning <5000 THB per month (32.13%), while most health science students reported incomes between 5001–10000 THB (50.92%).

Family smoking history was more prevalent among non-health science students (45.85%) compared to health science students (32.52%) (p=0.006). Similarly, alcohol use was significantly higher among non-health science students (89.17%) than health science students (72.39%) (p<0.001).

E-cigarette use was also more common among non-health science students (29.96%) compared to health science students (15.34%), with a statistically significant difference (p=0.001). While the gender distribution showed a slightly lower proportion of males in health science faculties (25.15%) compared to non-health science faculties (29.60%), this difference was not statistically significant (p=0.32).

### Comparison of knowledge about e-cigarettes between health science and non-health science students

Table 2 presents the comparison of e-cigarette knowledge between health science and non-health science students. No significant differences were observed in the perception of whether e-cigarettes are legally sold (AOR=1.12; 95% CI: 0.81–1.56; p=0.49), whether possession is illegal (AOR=1.00; 95% CI: 0.75–1.32; p=0.99), or whether e-cigarettes are addictive (AOR=1.04; 95% CI: 0.69–1.58; p=0.83). However, health science students were significantly more likely to correctly identify that e-cigarettes can cause lung inflammation (AOR=0.69; 95% CI: 0.47–0.98; p=0.04) and that they are associated with lung cancer risk (AOR=1.62; 95% CI: 1.14–2.28; p=0.006). In contrast, non-health science students were more likely to believe that e-cigarettes are more effective for smoking cessation than nicotine replacement therapies (AOR=1.36; 95% CI: 1.05–1.77; p=0.02). No significant group differences were observed in knowledge regarding e-cigarette use on airplanes (AOR=0.86; 95% CI: 0.45–1.64; p=0.64) or the ability to self-adjust nicotine dosage (AOR=0.96; 95% CI: 0.76–1.24; p=0.80).

**Table 2:** Comparison of knowledge about e-cigarettes between health science and non-health science students, a cross-sectional study at Ubon Ratchathani University, Thailand, 2023 (N=440)

*Question*	*Answer*	*Health science* *n (%)*	*Non-health science* *n (%)*	*p*	AOR*
1. E-cigarettes are legally sold products	Incorrect	136 (83.44)	236 (85.20)	0.49	1.12 (0.81–1.56)
	Correct	27 (16.56)	41 (14.80)		
2. Possession of e-cigarettes is not illegal	Incorrect	122 (74.85)	207 (74.73)	0.99	1.00 (0.75–1.32)
	Correct	41 (25.15)	70 (25.70)		
3. E-cigarettes are not addictive.	Incorrect	149 (91.41)	251 (90.61)	0.83	1.04 (0.69–1.58)
	Correct	14 (8.59)	26 (9.39)		
4. E-cigarettes can cause lung inflammation	Incorrect	14 (8.59)	39 (14.08)	0.04	0.69 (0.47–0.98)
	Correct	149 (91.41)	238 (85.92)		
5. E-cigarettes contain no nicotine	Incorrect	149 (91.41)	240 (86.64)	0.42	1.16 (0.80–1.69)
	Correct	14 (8.59)	37 (13.36)		
6. E-cigarettes do not cause lung cancer	Incorrect	148 (90.80)	226 (81.59)	0.006	1.62 (1.14–2.28)
	Correct	15 (9.20)	51 (18.41)		
7. People exposed to e-cigarette vapor are not considered secondhand smokers	Incorrect	144 (88.34)	222 (80.14)	0.09	1.33 (0.96–1.86)
	Correct	19 (11.66)	55 (19.86)		
8. E-cigarettes are more effective at helping people quit smoking than nicotine replacement therapies	Incorrect	128 (78.53)	168 (60.65)	0.02	1.36 (1.05–1.77)
	Correct	35 (21.47)	109 (39.35)		
9. E-cigarettes can be used on airplanes	Incorrect	158 (96.93)	267 (96.36)	0.64	0.86 (0.45–1.64)
	Correct	5 (3.07)	10 (3.61)		
10. E-cigarettes allow users to adjust the nicotine dosage themselves	Incorrect	66 (40.49)	107 (38.63)	0.80	0.96 (0.76–1.24)
	Correct	97 (59.51)	170 (61.37)		

* Reference category is ‘Incorrect’. Adjusted odds ratios (AORs) and 95% confidence intervals (CIs) were obtained from multivariate logistic regression, adjusted for age, sex, income, family smoking history, alcohol use, and e-cigarette use.

### Comparison of attitudes toward e-cigarettes between health science and non-health science students

Table 3 compares student attitudes toward e-cigarettes. No significant differences were found in the belief that e-cigarettes are safer than traditional cigarettes (AOR=1.04; 95% CI: 0.75–1.44; p=0.80), represent modernity or global trends (AOR=1.04; 95% CI: 0.75–1.45; p=0.82), or have appealing design and aesthetics (AOR=0.88; 95% CI: 0.68–1.13; p=0.32). However, non-health science students were significantly more likely to believe that e-cigarettes reduce the risk of smoking-related diseases, such as lung cancer (AOR=1.74; 95% CI: 1.77–2.55; p=0.005). No significant differences were observed in attitudes regarding e-cigarettes facilitating social integration (AOR=1.17; 95% CI: 0.82–1.65; p=0.39) or accessibility via retail and online platforms (AOR=1.12; 95% CI: 0.75–1.65; p=0.58). Both groups also held similar views on e-cigarette vapor being more pleasant than traditional cigarette smoke (AOR=0.94; 95% CI: 0.73–1.21; p=0.63).

**Table 3:** Comparison of attitudes toward e-cigarettes between health science and non-health science students, a cross-sectional study at Ubon Ratchathani University, Thailand, 2023 (N=440)

*Question*	*Answer*	*Health science* *n (%)*	*Non-health science* *n (%)*	*p*	AOR*
1. The use of electronic cigarettes (e-cigarettes) is perceived to be safer than traditional cigarettes	Disagree	140 (85.89)	219 (79.06)	0.80	1.04 (0.75–1.44)
	Agree	23 (14.11)	58 (20.94)		
2. Using e-cigarettes may contribute to harm reduction by decreasing the use of other psychoactive substances	Disagree	151 (92.64)	230 (83.03)	0.14	1.33 (0.91–1.94)
	Agree	12 (7.36)	47 (16.97)		
3. E-cigarette use is often associated with modernity and being up-to-date with global trends	Disagree	140 (85.89)	230 (83.03)	0.82	1.04 (0.75–1.45)
	Agree	23 (14.11)	47 (16.97)		
4. The design and aesthetics of e-cigarettes enhance their user appeal	Disagree	99 (60.74)	166 (59.93)	0.32	0.88 (0.68–1.13)
	Agree	64 (39.26)	111 (40.07)		
5. E-cigarettes are believed to lower the risk of smoking-related diseases, such as lung cancer	Disagree	153 (93.87)	229 (82.67)	0.005	1.74 (1.77–2.55)
	Agree	10 (6.13)	48 (17.33)		
6. E-cigarettes facilitate social integration and peer acceptance	Disagree	149 (91.41)	225 (81.23)	0.39	1.17 (0.82–1.65)
	Agree	14 (8.59)	52 (18.77)		
7. E-cigarettes are accessible through numerous retail and online distribution channels	Disagree	23 (14.11)	26 (9.39)	0.58	1.12 (0.75–1.65)
	Agree	140 (85.89)	2251 (90.61)		
8. E-cigarette vapor produces a pleasant aroma, which is less offensive than the odor of traditional cigarette smoke	Disagree	106 (65.03)	161 (58.12)	0.63	0.94 (0.73–1.21)
	Agree	57 (34.97)	116 (41.88)		

* Reference category is ‘Agree’. Adjusted odds ratios (AORs) and 95% confidence intervals (CIs) were obtained from multivariate logistic regression, adjusted for age, sex, income, family smoking history, alcohol use, and e-cigarette use.

## DISCUSSION

This study revealed significant knowledge gaps and misconceptions about e-cigarettes across both student groups, despite health science students exhibiting better awareness of health risks. Although global use of conventional tobacco has declined in the past decade, largely due to stringent regulations and sustained public health initiatives^2^. However, the rise of e-cigarettes presents a significant challenge to achieving smoke-free goals^20^. E-cigarettes, often marketed as safer alternatives, are increasingly popular among youth. In Thailand, where the importation, sale, and use of e-cigarettes remain banned, their consumption persists. Earlier studies reported prevalence rates of 20.4% ever using e-cigarettes^16^. This study reveals a record-high prevalence of 24.54% among undergraduate university students, highlighting the rapid upward trend of e-cigarette use, challenging the effectiveness of existing bans. Although Thailand’s prohibition aims to safeguard public health, these findings suggest that legal restrictions alone may fail to deter usage, potentially encouraging illicit markets^20^. In contrast, studies from more permissive contexts indicate that regulated approaches can guide safer use and support harm reduction^21^.

The non-health science student group showed a higher percentage of e-cigarette users. This finding is consistent with a previous study, which indicates that students from non-health-related faculties may be less aware of potential risks associated with using e-cigarettes^22^. Our study further supports this finding, showing that majority of participants misunderstood key domains including laws, health effects, and addiction nature, with no significant differences in overall knowledge between health and non-health science students. However, health science students had significantly better knowledge in the health effects domain, particularly regarding risks like lung injury.

This study found that over 60% of participants believed e-cigarettes to be more effective than nicotine replacement therapies (NRTs) for smoking cessation, revealing a notable misconception. While some studies have reported potential benefits of e-cigarettes in aiding smoking cessation, particularly under regulated conditions, the World Health Organization (WHO) and the Framework Convention on Tobacco Control (FCTC) Conference of the Parties (COP) advise against their promotion. These bodies emphasize the lack of conclusive evidence on long-term safety and effectiveness and call for precautionary approaches and regulatory control to prevent unintended consequences, especially among youth and non-smokers^23^. In contrast, one meta-analysis reported that e-cigarettes do not consistently yield higher cessation rates and may encourage dual use with traditional cigarettes^24^. E-cigarette aerosols contain nicotine, volatile organic compounds, and other potentially carcinogenic substances. While often viewed as less harmful than conventional cigarettes, e-cigarettes are not without risk^25^. Importantly, our study found a statistically significant difference, as health science students answered incorrectly more frequently than their non-health science students with respect to the efficacy of e-cigarettes. This is particularly concerning because healthcare students are future professionals who will provide patients with guidance and support regarding smoking cessation. These results highlight the crucial necessity of enhancing healthcare students’ education on the evidence-based information of e-cigarettes. It is essential to highlight that research findings remain inconclusive on the efficacy of e-cigarettes in facilitating smoking cessation to ensure that they can offer appropriate patient guidance.

Our study demonstrating predominantly negative attitudes toward e-cigarettes among Thai university students, regardless of their academic discipline, outlines the potential influence of stringent regulations on public perceptions. Thailand, as a nation that has implemented comprehensive bans on e-cigarettes, can serve as a valuable reference point for other jurisdictions considering or refining their regulatory approaches. The consistently negative attitudes observed in this study contrast with patterns in countries like the United Kingdom and the United States, where more balanced regulatory approaches have contributed to greater acceptance and a more nuanced understanding of e-cigarettes^21,26,27^. The study from Gravely et al.^21^ reported significant variation in e-cigarette awareness, experimentation, and current use across 14 countries, with those having more permissive regulatory environments generally reporting higher prevalence. Their findings indicated that where marketing and sales of e-cigarettes were less restricted, smokers and ex-smokers were more inclined to try these products, potentially integrating them into harm reduction strategies. Conversely, more restrictive contexts saw lower usage, suggesting that policy frameworks strongly influence consumer behavior. Similarly, Pinho-Gomes et al.^26^ highlighted the need for adaptive, integrated tobacco control policies that account for emerging products, arguing that comprehensive, evidence-based approaches are critical for effectively managing nicotine use and improving public health outcomes. Previous studies conducted in Thailand also point to the influence of strict prohibitions in shaping perceptions, often leaving individuals without reliable health information and supportive cessation strategies^15-17^. These findings suggest that simply imposing bans may not achieve intended public health outcomes. Moving forward, policymakers could consider integrating evidence-based education, transparent labeling, and accessible cessation resources. Such measures may foster a more informed public discourse and encourage rational decision-making, thereby making regulatory efforts more responsive to evolving international contexts.

### Limitations

This study has several limitations that warrant consideration. Its cross-sectional design precludes the establishment of causal relationships. Additionally, the findings, based on data collected from a single university using convenience sampling, may not be generalizable. Furthermore, the illegal status of e-cigarettes in Thailand could have influenced participants’ willingness to disclose their actual usage, potentially introducing response bias.

## CONCLUSIONS

The findings of this study indicate that both health science and non-health science students exhibit inadequate knowledge and attitudes regarding e-cigarette use, particularly in relation to the health risks and benefits. While health science students demonstrated a stronger understanding of specific health risks such as lung inflammation and cancer, misconceptions persist, particularly regarding the role of e-cigarettes in smoking cessation. Non-health science students, on the other hand, were more likely to hold favorable attitudes toward e-cigarette use and its perceived benefits. These results suggest the need for targeted educational initiatives, social interventions, and policy measures aimed at improving awareness and discouraging e-cigarette use among both groups. Public health efforts should focus on providing accurate information about the health risks of e-cigarettes and addressing the misconceptions related to their safety and effectiveness in smoking cessation. Further research should evaluate the impact of these interventions on attitudes and behaviors related to e-cigarette use.

## Data Availability

The data supporting this research are available from the authors on reasonable request.
